# Development and Psychometric Assessment of the Problematic QQ Use Scale among Adolescents

**DOI:** 10.3390/ijerph18136744

**Published:** 2021-06-23

**Authors:** Jintao Liu, Md Zahir Ahmed, Oli Ahmed, Mark D. Griffiths, Lili Chen

**Affiliations:** 1 Research Center for Urban Social Psychology, School of Education, Lanzhou City University, Lanzhou 730070, China; 2 School of Psychology, Northwest Normal University, Lanzhou 730070, China; ahmedzahirdu@gmail.com (M.Z.A.); chenlili.nwnu@gmail.com (L.C.); 3 Department of Psychology, University of Chittagong, Chattogram 4331, Bangladesh; oliahmed_polash131@cu.ac.bd; 4 Psychology Department, Nottingham Trent University, Nottingham NG1 4FQ, UK; mark.griffiths@ntu.ac.uk

**Keywords:** social media use, problematic QQ use, Problematic QQ Use Scale, psychometrics

## Abstract

The QQ social media platform is very popular among Chinese adolescents. As with other social media platforms (e.g., Facebook, Instagram, YouTube, etc.), there have been increasing reports that the use of QQ can be potentially problematic to a minority of users. However, unlike these other social media platforms, there is currently no scale to assess the risk of problematic QQ use. The present study developed the Problematic QQ Use Scale (PQQUS) among Chinese adolescents based on six core criteria of behavioral addiction (salience, tolerance, mood modification, loss of control, withdrawal, and conflict) that have been used in the development of other social media addiction scales. The scale was administered to a sample of 1008 Chinese school children to assess its psychometric properties, utilizing both classical test theory and item response theory. The analysis demonstrated that the PQQUS had good item discrimination indices relating to both CTT and IRT. The CFA results and Loevinger’s *H*-coefficient suggested the PQQUS had a unidimensional factor structure. The PQQUS had good internal reliability, good composite reliability, and good concurrent validity (based on correlations with measures of anxiety, depression, self-esteem, and life satisfaction). The invariance testing between boys and girls suggested this scale is a valid assessment tool for both groups. Overall, the PQQUS is a psychometrically robust tool for assessing problematic QQ use and will have a key role in further research on problematic QQ use among Chinese adolescents.

## 1. Introduction

Traditionally, addiction as a construct was only confined to substance abuse, but more recently, the construct has been applied to excessive problematic behaviors such as internet use, shopping, work, exercise, gambling, gaming, and social media [1,2,3,4,5]. Although behavioral addiction is a controversial concept, these problematic behaviors (gambling, excessive use of the internet, gaming, shopping, sex, and eating) have been recognized as non-substance addictions [1,6,7,8,9]. The outcomes of both substance and non-substance (i.e., behavioral) addictions appear to have similar effects, both behaviorally and psychologically [5]. Despite the growing use of the term ‘addiction’ to describe such activities, there is much debate over the term when applied to behaviors [1]; therefore, the present paper generally uses the term ‘problematic’ to describe behaviors that many scholars view as addictive.

A more recent problematic online behavior that has started to be investigated is problematic social media use along with its subvarieties such as problematic Facebook use [10], problematic Instagram use [11], problematic Tinder use [12], problematic Twitter use [13], and problematic YouTube use [14]. In some instances, some types of social media use (e.g., social networking) are used to maintain offline networking and relationships, whereas for some individuals, all their social networking is online. However, in a minority of cases, such maintenance can often lead to the excessive or problematic use of social media [3]. 

The relationship between social media use and mental health has been studied extensively over the past decade. Excessive use of social media has been associated with both anxiety and depression [15,16], and these associations are likely to be bidirectional. There are multiple reasons why individuals with anxiety and depression use social media excessively (and in some instances, problematically). One such reason for the association is the experience of self-gratification and relief from negative feelings [17] because it allows the user to escape from unpleasant and negative emotions [18]. For seeking pleasure and alleviating pain, individuals with anxiety and depression are more likely to have low self-regulation and higher internet use expectancies, leading to the excessive use of social media. 

One of the reasons why excessive use of social media has a probable association with depressive symptoms is the fact that excessive and problematic social media use can cause sleep-related problems (e.g., insomnia), which can be a factor in elevated depression [19,20,21]. Previous research has found that self-esteem is negatively related to problematic internet use [22]. Unrestrained use of social media can reduce face-to-face social interactions, facilitate loneliness, and lower self-esteem [23]. Self-esteem and life satisfaction have been reported as being predictors of problematic internet use [24]. Alexander argued that the physical disintegration of societal aspects has the potential to facilitate problematic internet use or addiction [25]. Problematic internet use often leads to isolation and withdrawal from societal processes, where users prefer online socialization rather than physical connectivity [26]. Social incoherence has been shown to be associated with addictive behavior [25,27,28]. 

A study on problematic Facebook use found that it is negatively associated with self-esteem [29]. According to the ‘social compensation hypothesis’ [30], individuals who have low self-esteem, are lonely, have less social support, and experience social anxiety may compensate for their offline social relationship difficulties through online communication. A negative association has also been found between problematic Facebook use and life satisfaction [29]. Excessive Facebook use has also been found to have detrimental effects on life satisfaction when users replace offline social relationships with online ones [31]. 

### 1.1. The QQ Social Media Platform in China

QQ, one of the largest social media platforms in China, has been highly popular for many years. The most recent report estimated that there were 639 million active QQ users in March 2021, which is more than the number of Twitter users around the world [32]. The number of internet users in China is over 872 million and it is estimated to reach one billion users by 2022 [33]. The prevalence of Chinese students’ problematic smartphone use is the highest compared to other samples from Asia [34], and QQ (typically accessed via smartphone) is one of the most frequently used social media and instant messaging services since 1999 [35]. A study of Chinese students reported that the abrupt change from a rigorously controlled secondary school entity to a more unrestricted college experience affected their smartphone use due to the prolonged time spent [36].

Adolescents are significantly more likely to engage in extreme or disruptive smartphone usage than adults [37]. The behavioral well-being of adolescents has been reported to be adversely associated with problematic smartphone use. The existing literature has shown that problematic smartphone use among adolescents is a potential cause for concern: 10% in the United Kingdom [38], 16.7% in Taiwan [39], 16.9% in Switzerland [40], 30.9% for Korea [41], and 31% in India [42]. In a South Korean study, 80.4% of participants at elementary schools started using smartphones at the age of 10 years or younger, and 59.9% of the participants used their smartphones for at least one hour or more every day [43]. In China, the average prevalence of problematic internet usage among adolescents was reported at 26.50% in one study [44], which is significantly higher than any other Asian countries (between 6.2% and 21.2%) [45]. In addition, problematic internet use has been associated with psychological conditions in children, such as anxiety, attention deficit disorder, depression, substance abuse disorder, and hyperactivity [46]. Adolescents who are problematic internet users often have a lack of offline social interactions compared to non-problematic users [47]. Furthermore, among adolescents, problematic internet usage has been shown to have a detrimental effect on sleep quality [48], health status, and quality of life [49]. 

In recent years, a number of studies have investigated problematic internet use and its association with other variables among the Chinese adolescent population. The most commonly reported risk factors for Chinese adolescent problematic internet use include poor sleep quality, anxiety, depression, and obesity [50,51,52]. Although there have been a number of studies [39,44,45,46,47,48,49] examining the consequences of problematic internet use (including problematic social media use) among students and adolescents in China, there is no psychometric instrument that has been developed to assess problematic QQ use, even though it is one of the most frequently used social networking platforms. 

As a specific social media application, this Chinese social networking platform has distinctive features (including a wallet (a mobile payment product incorporating multiple payment methods such as bank card payment, QR code payment, etc.), instant messaging, blog facilities, video sharing, and online gaming), which may have different relationships and associations with some psychological risk factors (e.g., depression, anxiety, life satisfaction, self-esteem) [53]. Research has shown that problematic internet use can lead to various adverse repercussions, including poor academic performance, anxiety, depression, insomnia, declining parental and friend relationships, and drug abuse [54]. Problematic social media use has been associated with mental disorders, including depression [55], insomnia [56], attention deficit, social phobia [57] and hyperactivity disorder [58]. Carli et al. [59] reported that individuals who use their smartphones and the internet in a problematic way are more likely to have medical comorbidities than non-problematic users. Any particular social media platform that offers a substantial number of features (including payment gateway, media sharing, gaming, etc.) increases the likelihood of individuals using that platform for a prolonged time duration, leading to problematic internet and smartphone use among a minority of users. 

### 1.2. The Present Study

Given that QQ is one of the most popular social media platforms in China and the relationship between its problematic use and anxiety, depression, self-esteem, and life satisfaction has been rarely studied, the present study examined these relationships. Moreover, although there are many psychometric instruments that have been developed to assess problematic social media use on platforms such as Facebook, YouTube, Tinder, and Instagram, as aforementioned, there is no such psychometric scale that assesses the risk of problematic QQ use. Therefore, the present study developed the Problematic QQ Use Scale (PQQUS) and investigated the relationship between problematic QQ use and mental health (i.e., anxiety, depression, self-esteem, and life satisfaction) among Chinese adolescents (who are particularly heavy users of the platform in China). The specific objectives of this present study were to: (i) assess the construct validity of the PQQUS, (ii) assess the reliability of the PQQUS, and (iii) assess the relationships between PQQUS scale scores, and anxiety, depression, life satisfaction, and self-esteem. Apart from the specific objectives, the general hypotheses of the present study were that the PQQUS would have: good discrimination index (H_1_); good construct validity in assessing problematic QQ use (H_2_); good reliability in assessing problematic QQ use (H_3_); significant associations with anxiety, depression, self-esteem, and life satisfaction (H_4_); and scalar strict level measurement invariance between boys and girls (H_5_).

## 2. Method

### 2.1. Participants and Procedure

In the present study, a cross-sectional survey design was utilized to test the formulated hypotheses. Chinese middle and high school going students were the target population of the present study. A total of four schools in the northwest region of China were selected utilizing a convenience sampling technique to recruit the participants. With a statistical power of 0.99 (*α* = 0.01) to detect the small-sized correlation coefficient (0.20) [60], a minimum number of 588 participants was required. A total of 1008 student participants (514 males [51%] and 494 females [49%]) were recruited from the four schools. The participants’ age range was 11–17 years (mean = 13.14 years; *SD* = 1.00 years). Data were collected in school classrooms from the participants using a questionnaire booklet that included demographic questions and the scales listed below. Each booklet took approximately 20 min to complete. One of the present authors and a group of research assistants distributed the booklet to approximately 1200 participants. A total of 1008 participants returned their completed booklet (response rate = 82%). One month was taken to collect all the data. 

### 2.2. Measures

In the present study, the survey contained a total of 67 questions including demographic information (three questions), problematic QQ use (six questions), anxiety (21 questions), depression (21 questions), self-esteem (10 questions), and life satisfaction (5 questions). The survey included the following scales (all details below)—the Problematic QQ Use Scale (PQQUS), Beck Anxiety Inventory (BAI: [61]; Chinese version: [62]), Beck Depression Inventory (BDI-II: [63]; Chinese version: [64]), Rosenberg Self-esteem Scale (RSES: [65]; Chinese version: [66]) and Satisfaction with Life Scale (SLS: [67]; Chinese version: [68]) along with a demographic information sheet (gender, age, number of family members, etc.). 

#### 2.2.1. Problematic QQ Use Scale

In developing the Problematic QQ Use scale, several steps were taken. First, the relevant literature concerning problematic online behavior and problematic internet/Facebook/social media use symptoms were examined. Additionally, psychometric scales used to assess problematic social media use, problematic internet use, and problematic smartphone use were investigated and reviewed. Many of these instruments were based on the ‘addiction components model’ [1], which posits that behavioral addictions comprise six core criteria (salience, tolerance, mood modification, loss of control, withdrawal, and conflict). Consequently, six items for assessing problematic QQ use were based on the item structure of the Bergen Facebook Addiction Scale (BFAS: [10]) and the Bergen Social Media Addiction Scale (BSMAS: [69]). These six items (see Appendix A for the six items) were adapted to QQ (‘Spent a lot of time thinking about QQ or planned use of QQ?’, ‘Felt an urge to use QQ more and more?’) and were piloted in interviews with of a convenience sample of 20 adolescents. These items were adjusted until they were clear. Once there were no problems with participants’ understanding, the PQQUS was utilized in the final study. Higher scores on the PQQUS suggest higher problematic QQ use. All psychometric properties concerning the PQQUS are described in the ‘Results’ section.

#### 2.2.2. Beck Anxiety Inventory (BAI)

The BAI comprises 21 items that assess anxiety symptoms; more specifically, psychological, emotional, and cognitive symptoms of anxiety (BAI [61]; Chinese version [62]). Participants rate all items (‘Numbness or tingling’, ‘Feeling hot’) based on the past seven days on a four-point scale ranging from ‘0′ (*not at all*) to ‘3′ (*severely “I could barely stand it”*). The total scores range from 0 to 63, and a higher total score indicates greater anxiety. The present study utilized the following cut-off scores to assess anxiety level: 0–7 = no anxiety; 8–15 = mild anxiety; 16–25 = moderate anxiety; and 26–63 = severe anxiety [62]. In the present study, the BAI had excellent internal consistency reliability (*ω* = 0.942, and *α* = 0.941). 

#### 2.2.3. Beck Depression Inventory (BDI)

The BDI comprises 21 items that assess the severity of depression [70]. To be more consistent with the depression criteria suggested by the DSM-IV [71], the tool was revised in 1996 (BDI [63]; Chinese version [64]). To assess depression levels, participants are asked to respond to the items on a four-point scale from 0 to 3 (e.g., ‘I do not feel sad’ [scoring 0], ‘I feel sad’ [1], ‘I am sad all the time and I can’t snap out of it’ [2], and ‘I am so sad and unhappy that I can’t stand it’ [3]). The total scores range from 0 to 63, and a higher total score indicates greater depression. The present study utilized the following cut-off scores to depression level: 0–13 = no depression; 14–19 = mild depression; 20–28 = moderate depression; and 29–63 = major depression [64]. Utilizing exploratory factor analysis, two factor structures (somatic-affective factor and cognitive factor) of the Chinese BDI-II have been reported and are the same as the English BDI-II. In the present study, the BDI had excellent internal consistency reliability (*ω* = 0.915, and *α* = 0.913). 

#### 2.2.4. Satisfaction with Life Scale (SWSL)

The SWSL [67] (Chinese version [68]) comprises five items that assess personal satisfaction with life through a global cognitive judgmental perspective. The items (e.g., ‘In most ways my life is close to my ideal’, ‘The conditions of my life are excellent’) are responded to on a seven-point Likert-type scale ranging from 1 (*strongly disagree*) to 7 (strongly agree). The total scores range from 5 to 35, and higher scores indicate higher life satisfaction. The present study utilized the following cut-off scores for life satisfaction level: 31–35 = extremely satisfied, 26–30 = satisfied, 21–25 = slightly satisfied, 20 = neutral, 15–19 = slightly dissatisfied, 10–14 = dissatisfied, and 5–9 = extremely dissatisfied. In the present study, the SWLS had very good internal consistency reliability (*ω* = 0.868, and *α* = 0.866).

#### 2.2.5. Rosenberg Self-Esteem Scale (RSES)

The RSES [65] (Chinese version: [66]) comprises 10 items that assess global self-esteem. The items (e.g., ‘Overall, I am satisfied with myself’, ‘At times I think I am no good at all’) are responded to on a four-point Likert-type scale ranging from ‘1′ (*strongly disagree*) to ‘4′ (*strongly agree*). The total scores range from 10 to 40, and higher scores indicate higher self-esteem. In the present study, the RSES had good internal reliability (*ω* = 0.796, and *α* = 0.793).

### 2.3. Ethics

The present study was carried out in accordance with the Declaration of Helsinki [72]. In addition, this study was approved by the ethical committee of the Northwest Normal University, China (ERB no. 20200030, dated: 22 October 2020).

### 2.4. Statistical Analysis

In the present study, the PQQUS’s psychometric properties were assessed using both classical test theory (CTT) and item response theory (IRT). For CTT, item analysis (corrected item-total correlation, average item-total correlation, Cronbach’s alpha, McDonald’s omega, and split-half reliability), exploratory factor analysis (EFA), confirmatory factor analysis (CFA), and multigroup confirmatory factor analysis (measurement invariance between boys and girls) were performed. Data were randomly split into two halves for performing EFA and CFA. Additionally, average variance extracted (AVE), composite reliability, Ferguson’s delta, and standard error of measurement were also calculated. For IRT, dimensionality (Loevinger’s *H*-coefficient), local independence (Yen’s *Q3*-coefficient), and monotonicity were calculated using the *R* package *mokken* version 3.0.2. Next, the psychometric properties (item discrimination/slope parameter (*α*), category difficulty/threshold parameter (*b*), and test information curve) were assessed through the graded response model (GRM) utilizing the R package *mirt* version 1.32.1. Additionally, *Rho* coefficient reliability was calculated utilizing the mokken package. As well as the aforementioned statistical tests, Pearson product-moment correlation coefficients were utilized to assess the relationships between problematic QQ use scores and anxiety, depression, life satisfaction, and self-esteem. In the present study, IBM SPSS v26, JASP v0.13.1.0, Microsoft Office Excel 2019, and RStudio were utilized for data management and data analysis.

## 3. Results

### 3.1. Participants’ Description

The mean age of the participants was 13.14 years (*SD* = 1.00 years). Among participants, 51% were boys and 49% were girls. Other demographic data (e.g., number of family members, grade, and average daily use of QQ) of the participants are presented in Table 1.

### 3.2. Main Results

**Figure 1:**
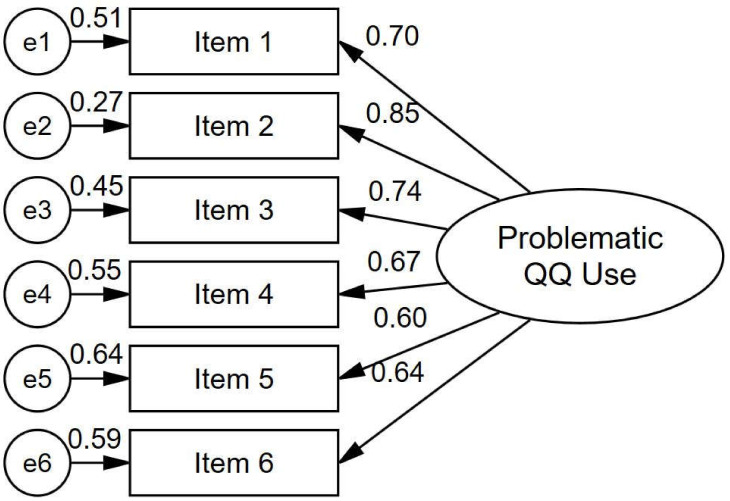
Factor structure and standardized loadings of the six items in the Problematic QQ Use Scale.

Table 2 shows the descriptive statistics (means and standard deviations, ranging between 1.48 (*SD* = 0.97) and 2.10 (*SD* = 1.16)) of the Problematic QQ Use Scale (PQQUS) along with item-level psychometric properties. All items in the scale had higher corrected item-total correlations ranging between 0.607 and 0.754. This suggests that all items had a good item discrimination index. The EFA results (Table 3) showed that the determinant value (0.076), KMO measure of sample adequacy (0.872), and Bartlett’s test of sphericity (*χ*^2^ = 1284.165. *p* < 0.001) were at acceptable levels. These values suggested the suitability of the data for performing EFA. EFA explored the unidimensional factor of the new developed scale (Eigenvalue = 3.60, variance extracted = 60.001). Factor loadings (Table 2) ranged between 0.71 and 0.84. The CFA results showed that the single-factor model (Figure 1) of the PQQUS yielded good model fits (*χ*^2^ = 20.574, df = 9, *p* = 0.015, *χ*^2^/df = 2.286, CFI = 0.987, TLI = 0.979, RMSEA = 0.051 [90% Lo = 0.021, Up = 0.080, *p* = 0.439], and SRMR = 0.068; Table 3). Factor loadings of the PQQUS (Table 2) ranged between 0.60 (Item 5 and Item 6) and 0.85 (Item 2). The aforementioned results of both EFA and CFA confirmed H_2_. 

Table 3 shows the scale-level psychometric properties of the PQQUS. Table 3 shows that the mean inter-item correlation (0.513) of the PQQUS was just above the recommended range (0.15–0.50). The PQQUS had good internal consistency reliabilities (Cronbach’s alpha = 0.863; McDonald’s omega = 0.866; and split-half reliability through the Spearman–Brown formula = 0.874). Table 4 also shows that the PQQUS had an acceptable average variance extracted value (0.60), composite reliability (0.90), and standard error of measurement (1.885 < *SD*/2 (2.546)). Additionally, the PQQUS had good discrimination power (Ferguson delta = 0.929). Results regarding reliabilities confirmed H_3_.

Statistics concerning the IRT assumptions (unidimensionality, local independence, and monotonicity) are presented in Supplementary Materials Table S1. The *H*-coefficient values for the PQQUS items ranged between 0.55 and 0.73. The PQQUS’s *H*-coefficient value was 0.552 (Table 3), which suggested that the scale under study was strongly unidimensional. The residual coefficients (Q3 coefficients) in Supplementary Materials Table S1 were also below the recommended cut-off (0.2) that ranged between −0.363 and 0.188. These values suggested the absence of local dependence between items. The monotonicity values in Supplementary Materials Table S1 show that violation of monotonicity, significant violation and *crit* values were zero for all of the items except Item 4. In Mokken scale analysis (MSA), crit is mentioned as an overall critical value for model violations [73,74]. Item 4 had two violations and one of these was significant (Supplementary Materials Table S1). However, the *crit* value of Item 4 was lower than the cut-off value (40), which suggested the absence of possible monotonicity. Therefore, all assumptions for applying an IRT model to analyze the PQQUS were met. In addition, the *Rho* reliability coefficient (0.867) of the PQQUS was above the recommended value (>0.7), which also suggested the good reliability of the scale.

**Figure 2:**
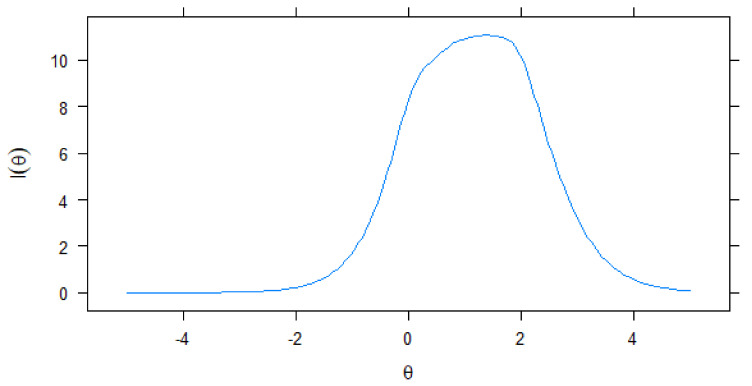
Test information curve of the Problematic QQ Use Scale.

Results regarding the graded response model of the IRT in Table 2 suggested that all items had very high item discrimination that ranged between 1.820 (Item 6) and 3.638 (Item 2) (mean *α* = 2.389). These item discrimination results confirmed H_1_ under the item response theory approach. Results regarding the *b*-coefficients in Table 2 suggested that a higher latent trait or theta was required to endorse all items, except Item 1. For instance, all the *b*-coefficients between Item 2 and Item 6 were positive, which suggests that an above average level theta is required to endorse Likert-type response categories between Items 2 and 5. In contrast to these items, a relatively lower latent trait or theta was required to endorse Item 1 (*b*-coefficients ranged between −0.320 and 2.060). These *b*-coefficients in Table 2 and the test information curve (Figure 2) suggested that the PQQUS was efficient in assessing average to higher levels of problematic QQ use. The test information curve showed that the PQQUS provides more information about individuals between 0 and 2.5 Θ level.

Results of the multigroup confirmatory factor analysis (Table 4) suggested the strict invariance between boys and girls. The configural model of the PQQUS had good model fit (*χ*^2^/df = 1.247, CFI = 0.997, RMSEA = 0.022, and SRMR = 0.053). The values in Table 5 show negligible changes in *χ*^2^ (non-significant *p*-value), CFI, RMSEA, and SRMR between configural and metric (1.852 [0.869], −0.002, −0.011, and 0.002, respectively). Changes in these values between metric to scalar (1.513 [0.911], −0.001, −0.011, and −0.006, respectively), and between scaler and strict (0.763 [0.993], 0, 0, and 0.001, respectively) were also negligible. Results regarding measurement invariance of the newly developed scale confirmed H_4_. These results suggested that the PQQUS is a valid measure for both boys and girls and assesses the same construct for both groups.

Table 5 shows the association of the PQQUS’s scores with per day QQ usage duration, anxiety, depression, self-esteem, and life satisfaction. The PQQUS scores positively and highly correlated with per day QQ usage duration (*r* = 0.602, *p* < 0.001, 95% CI [0.547, 0.657]), and moderately correlated with anxiety (*r* = 0.417, *p* < 0.001, 95% CI [0.365, 0.467]) and depression (*r* = 0.318, *p* < 0.001, 95% CI [0.261, 0.372]). The PQQUS scores also moderately but negatively correlated with self-esteem (*r* = −0.333, *p* < 0.001, 95% CI [−0.386, −0.276]) and life satisfaction (*r* = −0.327, *p* < 0.001, 95% CI [−0.381, −0.270]). These correlation coefficients suggested the concurrent validity of the PQQUS. These results regarding correlations confirmed H_5_.

## 4. Discussion

Excessive internet use and problematic internet use have been found to be key associated factors with poor mental health outcomes among a minority of adolescents. Mental health problems (e.g., depression, insomnia, anxiety increases, and social relationship weakness) have been found to positively correlate with problematic internet use [75]. Behavioral researchers have developed many psychometric tools to screen for the physical and psychological effects of problematic internet use and its many sub-varieties (such as social media addictions). The use of the internet in China is highly prevalent [20] and children and adolescents are spending increasing amounts of time online because education and entertainment (such as the use of QQ) and are becoming increasingly technology-dependent [76]. Consequently, the present study outlined the development of a new psychometric scale—the Problematic QQ Use Scale (PQQUS), which was developed to assess problematic QQ usage among Chinese adolescents. 

Results suggested that items of the newly developed PQQUS had a good discrimination index (corrected item-total correlation). This specific psychometric property indicated that the PQQUS differentiates between low scores and high scores on the scale. Results from both exploratory factor analysis (EFA) and the confirmatory factor analysis (CFA) suggested the factor structure of the newly developed scale was unidimensional. These results also indicated that the PQQUS had good construct validity. Results regarding the internal consistency reliability and composite reliability suggested that the PQQUS had good reliability in assessing the risk of problematic QQ use among Chinese adolescents. The multigroup CFA results suggested that the PQQUS assesses the same construct for both Chinese boys and girls. Overall, the results utilizing CTT suggested the PQQUS is a psychometrically robust instrument. 

The finding of unidimensionality supported the single-factor structure suggested by EFA and CFA. The graded response model’s results showed that the PQQUS performed adequately in assessing the risk of problematic QQ use among Chinese adolescents. All items of the PQQUS provided good differentiation information between individuals having different levels of the latent trait (i.e., problematic QQ use). Larger discrimination values of items also suggested that these items were highly related to the latent trait. Among the scale items, ‘conflict’ provided the least information, and ‘tolerance’ provided the most information. Items in the PQQUS were difficult in the sense that higher levels of problematic QQ use were needed for endorsement of the first category of most of the items. The test information curve suggested that the scale is efficient in assessing the risk of problematic QQ use among individuals having average to higher risk of problematic QQ use.

Scores on the PQQUS were positively associated with daily QQ usage duration, and scores for anxiety and depression, and negatively associated with scores for self-esteem and life satisfaction. A highly positive correlation between QQ usage duration and the Problematic QQ Use Scale suggested the validity of the score obtained via this scale. Previous studies have demonstrated the excessive use of social media is associated with higher levels of anxiety and depression and lower levels of self-esteem and life satisfaction [77,78]. On the one hand, individuals with high levels of depression and anxiety are more likely to have lower self-regulation and higher internet use expectancies [17], so they may be engaged with excessive QQ use and try to obtain more pleasure from QQ. On the other hand, sleep-related problems caused by excessive QQ use may lead to some mental health problems, such as depression. In addition, one of the reasons why individuals with low self-esteem are likely to get in the trouble in the real world and use QQ excessively may be that they need to compensate their problems in the real world by excessive QQ use. Likewise, individuals with lower life satisfaction might use QQ more excessively because they may be using QQ as a surrogate for happiness. Overall, the findings in the present study indicated that the PQQUS had good criterion validity. Furthermore, the study demonstrated that the PQQUS is a unidimensional scale with robust psychometric properties and that it is a good instrument to assess the risk of problematic QQ use among adolescents and among both genders. 

## 5. Limitations and Recommendations

Although this present study was statistically rigorous, it has several limitations. Firstly, the participants might have been biased with regard to social desirability and memory recall because the data were collected using a self-report survey. The use of the self-report to collect data has drawbacks. When individuals respond regarding their impressions, they are frequently discriminative in favor of them. This present study accepts the bias regarding the self-report approach, which could be avoided in future studies by using multi-method assessment. Secondly, the participants came from a narrow geographical location and the study was not nationwide, so the PQQUS’s reliability cannot necessarily be generalized to all Chinese adolescents. Given that the prevalence of problematic QQ use among adolescents, especially among school-going students, is unknown, a study with a large sample would complement the identification of the nature and prevalence of risk of problematic QQ use among Chinese adolescents. Furthermore, other problematic social media use (e.g., problematic WeChat use) could perhaps be assessed by adapting the PQQUS. Given the scale was validated using adolescents, further studies would be needed to see if the scale was psychometrically appropriate among other cohorts (e.g., adults).

## 6. Conclusions

The Problematic QQ Use Scale was developed among Chinese adolescents and was theoretically based on the six criteria for behavioral addiction suggested by Griffiths [1]. These criteria have been used in the development of other psychometric scales assessing various behavioral addictions (including problematic use of other social platforms). With multidimensional aspects, the concept of ‘addiction’ has expanded beyond that of the ingestion of psychoactive substances. Given that gambling in its most problematic form is now considered a bona fide addiction (and does not involve the ingestion of a psychoactive substance), there is no theoretical reason why other problematic behaviors that involve clinical impairment of individuals’ everyday lives such as various internet-related problematic use cannot be conceptualized as genuine addictions [1]. 

Problematic use of social media is an issue of increasing concern in China. However, there have been few specific psychometric tools developed to assess the risk of problematic use or addiction to Chinese-only social media platforms (e.g., QQ). The present study developed such a Chinese psychometric tool to screen for the risk of problematic QQ use among Chinese adolescents, comprising school-going students. Using convenience sampling and rigorous psychometric analysis, the present study demonstrated the PQQUS to be valid in assessing the risk of problematic use among Chinese adolescents. By utilizing the scale, mental health and other healthcare professionals in China can quickly, easily, and reliably assess adolescents’ risk of problematic QQ use. It is recommended that further research is conducted with larger and more nationally representative samples across different cohorts to explore all aspects of potential problematic QQ use in China.

## Figures and Tables

**Table 1:** Demographic distribution of participants.

Age	Mean (*SD*)	13.14 (1.00) Years
Gender	Boys	51%
	Girls	49%
Number of Family Members	2 Members	2.3%
	3 Members	45.2%
	4 Members	38.1%
	5 Members	11.2%
	6 Members	2.3%
	7 Members	0.6%
	8 Members	0.2%
	9 Members	1%
Grade	VI	8.2%
	VII	14.7%
	VIII	29.6%
	IX	16.4%
	X	11.2%
	XI	9.3%
	XII	10.6%
Time Spend on QQ	0–30 Minutes	55.7%
	31–60 Minutes	39.6%
	61-Above Minutes	4.7%

**Table 2:** Item-level psychometric properties of the Problematic QQ Use Scale.

Items	*M*	*SD*	Corrected Item-Total Correlation	Factor Loading		Graded Response Model				
				EFA	CFA	*α*	b_1_	b_2_	b_3_	b_4_
**Item 1**	2.10	1.16	0.630	0.74	0.70	2.255	−0.320	0.549	1.399	2.060
**Item 2**	1.83	1.15	0.754	0.84	0.85	3.638	0.146	0.814	1.326	1.872
**Item 3**	1.78	1.14	0.672	0.78	0.74	2.235	0.252	0.965	1.629	2.125
**Item 4**	1.72	1.16	0.648	0.78	0.67	2.336	0.386	1.137	1.582	1.968
**Item 5**	1.48	.97	0.632	0.79	0.60	2.049	0.765	1.567	1.999	2.421
**Item 6**	1.63	1.02	0.607	0.72	0.64	1.820	0.463	1.288	2.053	2.589

**Table 3:** Scale-level psychometric properties of the Problematic QQ Use Scale.

Psychometric Properties	Scores	Suggested Cut-Off
**Mean inter-item correlation**	0.513	Between 0.15 and 0.50
**Cronbach’s alpha**	0.863	≥0.7
**McDonald’s Omega**	0.866	≥0.7
**Split-half reliability (odd-even)**	0.874	≥0.7
**Average variance extracted**	0.60	≥0.5
**Composite reliability**	0.90	≥0.7
**Standard error of measurement**	1.885	Smaller than SD/2
**Ferguson delta**	0.929	≥0.9
**Loevinger’s *H*-coefficients**	0.552	-
***Rho* coefficient**	0.867	≥0.7
**Results of exploratory factor analysis**		
**Determinant**	0.076	>0.0001
**KMO measure of sample adequacy**	0.872	0.50
**Bartlett’s test of sphericity**	1284.17 (*p* < 0.001)	significant
**Eigen value**	3.60	1 or above
**Variance**	0.601	
**Model fits of confirmatory factor analysis**		
***χ*^2^ (df, *p* value), *χ*^2^/df**	20.574 (9, 0.015), 2.286	Nonsignificant, <5
***CFI***	0.987	>0.95
***TLI***	0.979	>0.95
***RMSEA* [90% CI value] (*p* value)**	0.051 [0.020, 0.080] (0.439)	<0.08
***SRMR***	0.068	<0.08

**Table 4:** Measurement invariance of the Problematic QQ Use Scale between boys and girls.

	*χ* ^2^	Df	Δ	Δdf	*p*	CFI	Δ	RMSEA	Δ	SRMR	Δ
**Configural Model**	22.446	18				0.997		0.022		0.053	
**Metric Model**	24.299	23	1.853	5	0.869	0.999	−0.002	0.011	−0.011	0.055	0.002
**Scaler Model**	25.812	28	1.513	5	0.911	1	−0.001	0	−0.011	0.049	−0.006
**Strict Model**	26.575	34	0.763	6	0.993	1	0	0	0	0.050	0.001

**Table 5:** Correlation coefficients of scores on the Problematic QQ Use Scale with anxiety, depression, self-esteem, and life satisfaction.

	QQ Addiction
**QQ usage duration**	*r* = 0.602, *p* < 0.001, 95% CI [0.547, 0.657]
**Anxiety**	*r* = 0.417, *p* < 0.001, 95% CI [0.365, 0.467]
**Depression**	*r* = 0.318, *p* < 0.001, 95% CI [0.261, 0.372]
**Self-esteem**	*r* = −0.333, *p* < 0.001, 95% CI [−0.386, −0.276]
**Life satisfaction**	*r* = −0.327, *p* < 0.001, 95% CI [−0.381, −0.270]

## Data Availability

The data presented in this study are available on request from the corresponding author.

## Supplementary Materials

The following are available online at https://www.mdpi.com/article/10.3390/ijerph18136744/s1, Supplementary Table S1: H-coefficient, Ǫ3 coefficient, monotonicity outputs of the QQ addiction scale.

## Appendix A

**Table A1:** English version: The Problematic QQ Use Scale for Adolescents.

SL	Item	Scoring				
		Very Rarely	Rarely	Sometimes	Often	Very Often
		1	2	3	4	5
1	Spent a lot of time thinking about QQ or planned use of QQ?	1	2	3	4	5
2	Felt an urge to use QQ more and more?	1	2	3	4	5
3	Used QQ in order to forget about personal problems?	1	2	3	4	5
4	Tried to cut down on the use of QQ without success?	1	2	3	4	5
5	Become restless or troubled if you have been prohibited from using QQ?	1	2	3	4	5
6	Used QQ so much that it has had a negative impact on your job/studies?	1	2	3	4	5
