# Supplementary material for: Development and Psychometric Assessment of the Problematic QQ Use Scale among Adolescents
Source: Int J Environ Res Public Health. 2021 Jun 23;18(13):6744. doi: 10.3390/ijerph18136744 (PMC8268458; doi:10.3390/ijerph18136744)
Supplement: Supplementary file 1 [file ijerph-18-06744-s001.zip › ijerph-1211599-supplementary.pdf]

Supplementary table

Supplementary Table S1: *H*-coefficient, *Q*3 coefficient, monotonicity outputs of the QQ addiction scale

	***H*- coefficients**	**Monotonicity**				**Yen's Q3-coefficients**				
		**#ac**	**#vi**	**#zsig**	***Crit***	**Item 1**	**Item 2**	**Item 3**	**Item 4**	**Item 5**
**Item 1**	.73	60	0	0	0					
**Item 2**	.73	47	0	0	0	-.031				
**Item 3**	.65	60	0	0	0	-.069	-.324			
**Item 4**	.59	60	2	1	21	-.275	-.259	-.160		
**Item 5**	.57	55	0	0	0	-.363	-.294	-.104	.006	
**Item 6**	.55	58	0	0	0	-.305	-.306	-.115	.023	.188

ac = active comparison, vi = violation, zsig = significant violation, Crit = overall critical value
